# Cross-sectional analysis of variation in diagnosis of Lewy body dementia in three English regions: data from the DETERMIND programme

**DOI:** 10.1136/bmjopen-2026-121991

**Published:** 2026-07-22

**Authors:** Calum A Hamilton, Alan J Thomas, Paul C Donaghy, Ben Hicks, Christoph Mueller, Sube Banerjee

**Affiliations:** 1 Translational and Clinical Research Institute, Newcastle University, Newcastle upon Tyne, UK; 2 Faculty of Medicine and Health Sciences, University of Nottingham, Nottingham, UK; 3 Institute of Psychiatry, Psychology and Neuroscience, King’s College London, London, UK; 4 South London and Maudsley NHS Foundation Trust, London, UK

**Keywords:** Dementia, Observational Study, Old age psychiatry, Cognitive dysfunction, NEUROLOGY

## Abstract

**Abstract:**

**Objectives:**

We aimed to examine regional differences in the relationship between core clinical features assessed using the Improving the DIAgnosis and Management Of Neurodegenerative Dementias of Lewy body type in the NHS (DIAMOND-Lewy) dementia with Lewy bodies (DLB) Assessment Toolkit and memory service diagnoses of Lewy body dementia (LBD).

**Design:**

Secondary analysis of a multicentre observational study.

**Setting:**

Memory clinics in three sites across England (North East, London and South East) from July 2019 to March 2023.

**Participants:**

935 individuals with a new memory service diagnosis of dementia enrolled in the DETERMinants of quality of life, care and costs, and consequences of INequalities in people with Dementia and their carers (DETERMIND) programme.

**Outcome measures:**

Core clinical features of DLB were assessed using the DLB Assessment Toolkit. The relationship between core clinical features and memory service diagnosis of LBD was examined using Bayesian probit models.

**Results:**

There were higher rates of LBD diagnosis from memory services in the cohort in North East England compared with the London and South East centres (11% vs 4%; risk ratio (RR)=1.72 (1.35–2.08)) and evidence of a regional moderating effect on the relationship between clinical features and LBD diagnosis (RR=1.73 (1.26–2.40)).

All core clinical features of DLB were associated with LBD diagnosis in North East England, whereas visual hallucinations were the most influential diagnostic feature in London and the South East (RR=3.18 (2.29–4.02)).

**Conclusions:**

Regional differences in LBD diagnosis in UK memory services appear to reflect different rates of recognition of specific LBD clinical features. Routinely using a standardised DLB Assessment Toolkit and improving awareness of non-hallucination features in LBD could help to address this disparity.

**STRENGTHS AND LIMITATIONS OF THIS STUDY**

- This study benefited from relatively large-scale screening of undifferentiated dementia cases to identify potentially unrecognised Lewy body dementia (LBD).
- The multisite setting enabled assessment of regional differences in disease prevalence and recognition.
- LBD diagnoses are treated probabilistically—we were unable to confirm these with biomarkers or gold-standard autopsy assessment.

## Introduction

 Lewy body dementias (LBD), including dementia with Lewy bodies (DLB) and Parkinson’s disease dementia (PDD), are the second most common neurodegenerative aetiologies in dementia according to gold-standard neuropathological studies, where rates of approximately 25% have been reported.^1^ In contrast, LBD is comparatively under-recognised in clinical practice, with DLB comprising only 4.6% of diagnosed dementia cases in the UK.^2^ This is consistent with evidence from highly sensitive α-Synuclein seed amplification assays which have shown positivity in 23% of individuals with mild cognitive impairment/dementia in the Swedish BioFINDER studies; of these α-Synuclein-positive individuals, only 21% had a DLB or PD clinical diagnosis.^3^ Taken together, this suggests that most cases of LBD may not be identified clinically.

Previous research has highlighted regional differences in the rate of diagnosis of LBD in the UK. Dementia services in North East England have a higher prevalence of DLB diagnosis than those in East Anglia.^2^ Regional disparities in diagnoses of Lewy body disease are not unique to the UK and have been described in Finland,^4^ Peru,^5^ Taiwan and other areas.^6^ As previously described,^2 7^ regional differences in diagnoses could reflect differences in true DLB rates due to varying exposure to environmental or other risk factors,^4 8–10^ varying rates of recognition of DLB due to local training and awareness, or a combination of these factors. If variations in LBD diagnosis are attributable to regional differences in recognition of LBD clinical features, then this could provide insights into how to improve diagnostic accuracy, and therefore improve access to appropriate management.^11^

PDD may be recognised by the presence of dementia in established Parkinson’s disease (PD),^12^ whereas DLB may be recognised by the presence of the core clinical features of: parkinsonism, rapid eye movement (REM) sleep behaviour disorder (RBD), cognitive fluctuations and visual hallucinations.^13^ The Assessment Toolkit for Dementia with Lewy Bodies was developed to help the recognition of these clinical features and consequently the diagnosis of DLB.^14^

This toolkit has been applied in various clinical and research settings, including within the DETERMIND (DETERMinants of quality of life, care and costs, and consequences of INequalities in people with Dementia and their carers) programme: an ongoing cohort study of people with newly diagnosed dementia, recruited from three English regions. Diagnoses were made by clinical services and all causes of dementia were eligible for inclusion in the cohort study. Participants were administered the DLB Assessment Toolkit as part of the research interview. Using the toolkit following diagnosis in different regions provides an opportunity to test whether regional differences in LBD diagnosis reflect true differences in disease prevalence or differences in disease recognition, by examining any mismatches between toolkit-identified LBD and clinical diagnoses of LBD made by memory services.

If there were true regional differences in LBD prevalence, then this should manifest as regional differences in frequency of toolkit-reported core clinical features. If instead regional differences in LBD diagnostic rates were due to differing recognition of LBD, then rates should be similar in toolkit-identified diagnoses across regions.

We therefore aimed to test: (1) whether UK regional disparities in LBD diagnoses were present in the DETERMIND cohort; (2) whether these were attributable to regional differences in true prevalence of latent LB disease, inferred from different regional rates of DLB clinical features identified using the assessment toolkit; and (3) whether there were regional differences in recognition of LBD given the clinical features present.

## Method

We conducted a cross-sectional analysis of baseline data from an ongoing longitudinal study of people recently diagnosed with dementia.

### Participants and procedure

Participants with dementia were recruited from three geographically and socially diverse areas in the UK—Gateshead (North East), South London and Sussex, Surrey and Kent (South East).^15 16^

Recruitment was predominantly from Memory Assessment Services and took place between July 2019 and March 2023 with a break from March 2020 to February 2021 due to the COVID-19 pandemic. People with dementia were eligible to participate providing they had received a clinical diagnosis of any dementia and consented onto the study within 6 months of receiving this diagnosis.

Where available, a carer was interviewed to provide additional information. People with dementia could participate in DETERMIND without a carer, while carers could only participate if approved by the person with dementia they were supporting.

Capacity to consent was assessed during face-to-face consultation by a trained researcher, and a personal consultee was sought for those people with dementia who lacked capacity to provide informed consent. Consent for the inclusion of a carer was sought from people with dementia with capacity and for those people without capacity, consent was sought from a personal consultee. All participants provided written, informed consent to participate.

Questionnaire data were collected predominantly through face-to-face interviews and typically lasted around two and a half hours at baseline. Questionnaires were designed to collect a range of data exploring postdiagnostic inequities and inequalities in care and services use. Study data were collected and managed using REDCap electronic data capture tools hosted at Yale University.^17^

### Measures

Clinical diagnosis of LBD was ascertained at study baseline. Participants were considered to have an LBD diagnosis if they had LBD, DLB or PDD as their dementia diagnosis. As this leveraged real-world LBD diagnoses, the specific diagnostic criteria used (if any) were not known.

The four core clinical features of DLB were ascertained through use of DLB Assessment Toolkit (see online supplemental table S1):

*Cognitive fluctuations* were identified through carer interview with four screening questions. Affirmative response to two or more screening questions was considered as suggestive of probable cognitive fluctuation, as recommended by the DLB Assessment Toolkit.^14^

*Visual hallucinations* were identified through interview with people with dementia and carers. Two screening questions were asked to people with dementia and carers, respectively. The DLB Assessment Toolkit recommends using clinical judgement to assess the presence of hallucinations according to these responses. In this study no such clinical judgement was available, so individuals were considered to have hallucinations if they answered affirmatively to either carer hallucination screening question:

Does [person’s name] have hallucinations such as seeing false visions?

or:

Does [person’s name] seem to see things that are not present?

or the second visual hallucination screening question administered to the person with dementia:

Have you ever seen something (or things) that other people could not see?

*Parkinsonism* was assessed using the 5-item Unified Parkinson’s Disease Rating Scale (UPDRS). Consistent with recommendations of the DLB Assessment Toolkit, a score of >7 on the 5-item UPDRS was considered suggestive of probable parkinsonism.

*REM sleep behaviour disorder* was assessed by a single screening question for dream enactment asked to carers:

Have you ever seen [person’s name] appear to ‘act out his/her dreams’ while sleeping (punched or flailed arms in the air, shouted or screamed)?

If a carer was not available to provide information on possible dream enactment, a secondary screening question was provided to the person with dementia themselves:

Have you ever been told that you seem to ‘act out your dreams’ while sleeping (punched or flailed arms in the air, shouted or screamed)?

### Analysis

All analyses were conducted using *R* statistical software for Windows (V.4.5.0) with the *brms* package as an interface to the *Stan* probabilistic programming language. Age and gender were included as covariates to account for possible systematic differences and the known male/female imbalance in DLB diagnoses.

LBD diagnostic rates were assessed with a Bayesian binomial probit model (codifying relationships with a binary variable in a comparable manner to a frequentist logistic regression and enabling features to be treated as ordinal predictors), including the four core clinical features specifically or total number of core features endorsed as predictors, with a binary site (North East vs London/South East, in the absence of any hypothesised London vs South East difference) interaction. Individual core clinical features were included as binary predictors, while the total number of features endorsed was treated as a monotonic ordinal variable.

Primary analyses were conducted with listwise deletion for missing data. Sensitivity analyses were also conducted with missing data multiply imputed using the *mice* package with predictive mean matching. All analytical variables (LBD diagnosis, region, age, gender and the four core clinical features of DLB) were included with an assumption that missingness was non-informative (ie, missing at random or missing completely at random, eg, reflecting unavailability of an informant which was more common for women). Posterior distributions were pooled by Bayesian methods.

Strength of evidence for each effect was interpreted with reference to the corresponding evidence ratio (ER; the weight of evidence for a given hypothesis: the weight of evidence against this) for each effect, equivalent to a Bayes factor for a directed hypothesis.^18^ ER values>1 reflect evidence in favour of the hypothesis, values<1 reflect more evidence against the hypothesis, while values≈1 reflect equivocal evidence. Bayesian analyses included weakly informative Student’s t priors with intercepts anticipating that LBD would represent a minority of dementia cases (ν=3, μ = −2, σ=1) and zero-centred coefficients for all predictor slopes (ν=8, μ=0, σ=1). Prior predictive checks and prior sensitivity analyses were used to check the robustness of results with different prior specifications.

### Patient and public involvement

A patient and public involvement group including experts by experience were involved in all stages of the DETERMIND research proposal and study conduct.

## Results

### LBD diagnostic rates in healthcare services

Of 935 participants, 57 (6%) had a clinical diagnosis of LBD: 35 of DLB and 22 PDD.

There was a higher rate of LBD diagnosis in North East England than London/South East overall: 11% of participants in North East England had received a diagnosis of LBD, compared with 4% of participants from London and the South East (see table 1). This provided strong evidence of a higher rate of LBD diagnosis in the North East versus South East/London (risk ratio (RR)=1.72 (1.35–2.08), ER=3999). There was evidence of a higher rate of LBD diagnoses in men (RR=1.48 (1.12–1.84), ER=307) and in those with a younger age at assessment (RR=0.979 (0.96–0.999), ER=54).

**Table 1:** Baseline characteristics of DETERMIND study participants by recruitment site

	Overall, N=935*	Gateshead (North East), N=258*	London, N=208*	Sussex (South East), N=469*	P value†
Lewy body dementia	57/925 (6.2%)	28/256 (11%)	7/207 (3.4%)	22/462 (4.8%)	<0.001
Lewy body dementia subtype					0.002
DLB	35/57 (61%)	20/28 (71%)	0/7 (0%)	15/22 (68%)	
PDD	22/57 (39%)	8/28 (29%)	7/7 (100%)	7/22 (32%)	
Age	81 (11)	83 (11)	79 (11)	81 (10)	0.004
Female gender	469/928 (51%)	145/257 (56%)	103/206 (50%)	221/465 (48%)	0.072
Visual hallucinations	237/645 (37%)	90/233 (39%)	36/104 (35%)	111/308 (36%)	0.732
Cognitive fluctuations	290/552 (53%)	111/199 (56%)	32/82 (39%)	147/271 (54%)	0.028
REM sleep behaviour disorder	99/308 (32%)	36/86 (42%)	17/55 (31%)	46/167 (28%)	0.068
Parkinsonism	45/654 (6.9%)	14/215 (6.5%)	3/81 (3.7%)	28/358 (7.8%)	0.403

* n/N (%); median (IQR).

† Pearson’s χ2 test; Fisher’s exact test; Kruskal-Wallis rank sum test.

DETERMIND, DETERMinants of quality of life, care and costs, and consequences of INequalities in people with Dementia and their carers; DLB, dementia with Lewy bodies; PDD, Parkinson’s disease dementia; REM, rapid eye movement.

### DLB core clinical feature rates identified using the DLB Assessment Toolkit

Person with dementia-reported and carer-reported rates of DLB core clinical features varied across sites (see table 1) and across specific items for each feature (see online supplemental table S1). However, age-adjusted and sex-adjusted models did not support any higher rates of cognitive fluctuations (RR=1.21 (0.808–1.71), ER=5), visual hallucinations (RR=1.12 (0.824–1.49), ER=3) or parkinsonism (RR=0.712 (0.156–2.76), ER=2) in North East England. There was evidence of higher carer-reported rates of RBD in North East England (RR=1.53 (1.09–2.0), ER=147), though patient-reported rates of RBD were similar across sites (see online supplemental table S1).

### Recognition of DLB according to number of features endorsed

**Figure 1:**
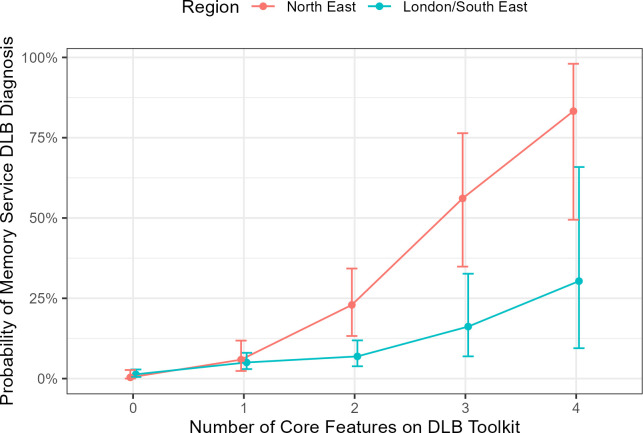
Estimated probability of having received a Lewy body dementia diagnosis in health services according to number of core clinical features identified by the Dementia with Lewy Bodies (DLB) Assessment Toolkit, with North East versus London/South East interaction.

In London/South East, there was a positive association between the number of DLB core features endorsed and the probability of having received an LBD diagnosis in memory services (RR=1.63 (1.30–2.05), ER>12 000). However, only a minority of individuals in London/South East endorsing 2–4 core features of LBD had received a memory service diagnosis of LBD (see figure 1).

This relationship was therefore strongly moderated by region with a positive interaction between number of features endorsed and being diagnosed in North East England (RR=1.73 (1.26–2.40), ER=1999), leading to greater LBD diagnosis rates for those with two or more core clinical features of DLB.

### Recognition of DLB according to specific features endorsed

In London and the South East, the presence of visual hallucinations was the strongest predictor of receiving an LBD diagnosis in healthcare services (RR=3.18 (2.29–4.02), ER>4000), followed by parkinsonism (RR=1.47 (1.08–1.7), ER=82). Presence of RBD (RR=2.83 (0.68–8.87), ER=13) or cognitive fluctuations (RR=1.04 (0.518–1.77), ER=1) were not as clearly associated with greater rates of reported LBD in clinical services.

**Figure 2:**
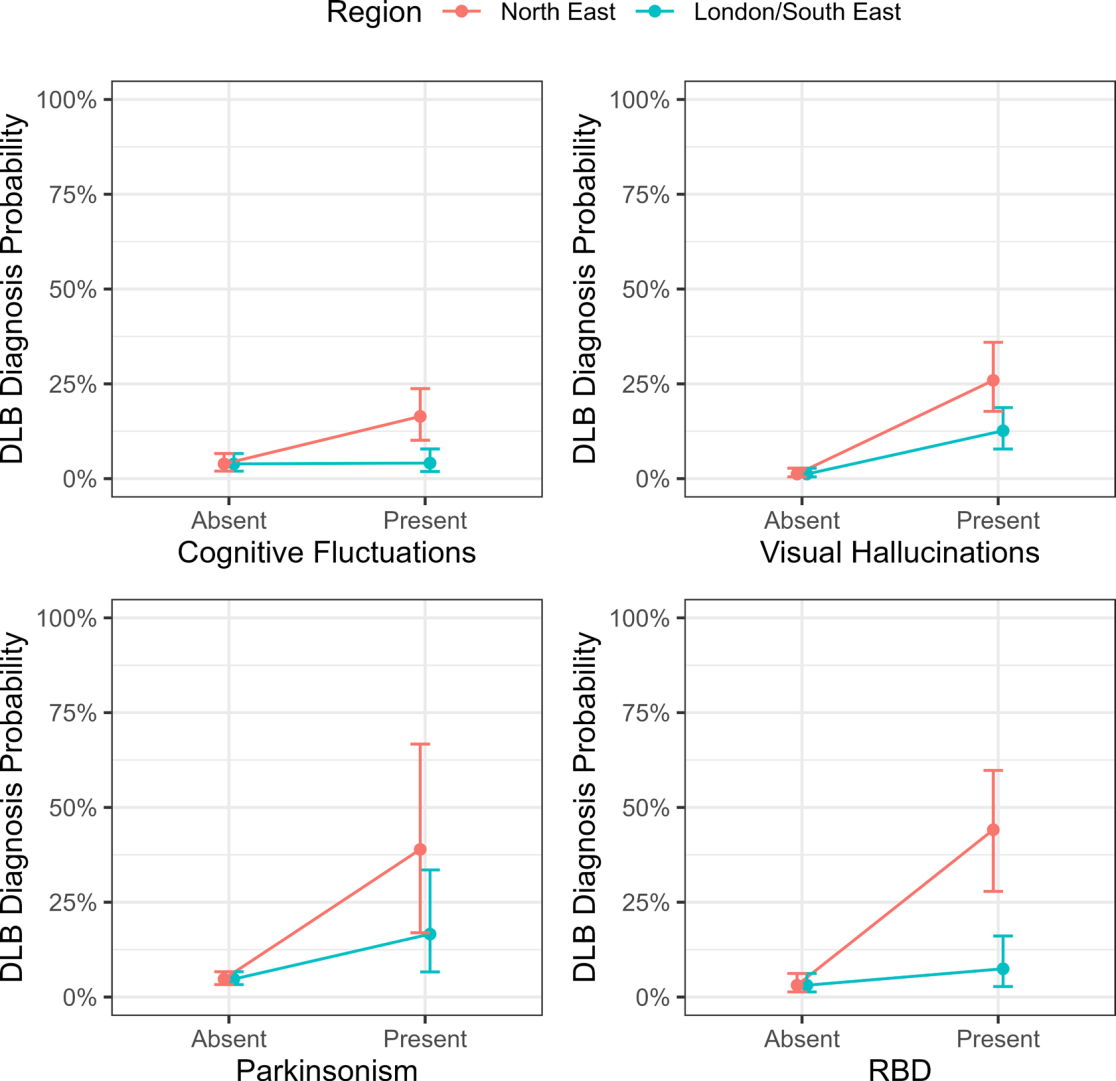
Estimated probability of dementia with Lewy bodies (DLB) diagnosis for individual core clinical features, with site interaction. RBD, rapid eye movement sleep behaviour disorder.

There was strong evidence that the higher rates of LBD diagnosis in North East England were due to a stronger recognition of Lewy body disease diagnostic features (see figure 2) of RBD (RR=14.9 (5.15–33.9), ER>4000), cognitive fluctuations (RR=2.46 (1.59–3.45), ER=1999) and visual hallucinations (RR=1.87 (1.16–2.68), ER=173) but not parkinsonism (RR=1.47 (0.884–1.78), ER=15).

Predicted probabilities of LBD diagnosis from multivariable models suggest that an exemplar individual with dementia and three core clinical features of DLB found using the Assessment Toolkit, but without visual hallucinations, had a 2.9% estimated probability of receiving a diagnosis of LBD in London/South East England. If they also reported visual hallucinations, this probability increased to 41.3%. In comparison, the same individuals had an estimated 81.1% and 95.1% probability, respectively, of being diagnosed with LBD by services in North East England.

### Sex differences in DLB clinical features and diagnoses

Adjusting for site level differences, there was no evidence for higher rates of any individual DLB core clinical features by sex: adjusting for site and age, cognitive fluctuations occurred in an estimated 54% of females vs 51% of males; visual hallucinations occurred in 36% of females vs 38% of males; parkinsonism occurred in 5% of females vs 7% of males; RBD was reported in 26% of females vs 34% of males.

There was also no clear evidence that sex moderated the relationship between DLB core features and LBD diagnoses (RR=0.70 (0.28–1.30), ER=6) or the relationship between site and LBD diagnosis (RR=0.46 (0.09–1.42), ER=9).

### Sensitivity analyses

There was no evidence of a difference in the number of DLB core clinical features identified between those with DLB versus PDD diagnoses (RR=1.0 (0.47 to 1.69), ER=1) or the prevalence of any specific DLB core clinical features identified by the assessment toolkit. Individual models predicting DLB and PDD provided comparable effect sizes but with wider credible intervals reflecting lower statistical power, supporting that the effect patterns did not differ between DLB and PDD. Analysis with multiple imputation for missing values provided comparable findings to the primary analyses.

## Discussion

In this multisite study of people with newly diagnosed dementia in the North East, London and South East England, we examined the possible mismatch between memory service diagnosed LBD (including DLB or PDD) and rates of core clinical features identified using the DLB Assessment Toolkit.

We found LBD was more often diagnosed in services in North East England than the other two regions of the UK studied, with less than half the rates of DLB diagnosis in London and the South East compared with the North East (~4% vs 11%), in line with previous findings of lower diagnostic rates in East Anglia versus the North East.^2^ The toolkit found similar rates of LB features across regions, which did not clearly support theorised regional differences in actual LB disease rates. Across all regions, LBD diagnostic rates were lower than expected from autopsy studies, and were low even in those with two or more core clinical features, making a case for routine use of the DLB Assessment Toolkit to improve diagnostic accuracy.

Our findings were therefore most consistent with these differences in LBD diagnoses reflecting regional differences in the clinical recognition of LBD core clinical features: both for specific features and their total accumulation. Regardless of region, individuals with more core features identified on the toolkit were more likely to have an LBD diagnosis. However, this effect was strongly moderated by site. Those with more DLB clinical features in the North East were substantially more likely to have a diagnosis of LBD, suggesting different vigilance, active identification and recognition of the clinical significance of multiple LB features in dementia in this region.

In London and the South East, presence of toolkit-assessed fluctuating cognition was not associated with higher likelihood of LBD diagnosis in memory services. In contrast, in North East England, individuals with toolkit-assessed fluctuating cognition had a higher likelihood of having received a diagnosis of LBD. This may reflect different (non-toolkit mediated) clinical practice due to higher awareness of LBD and therefore higher symptom vigilance in the North East. There was no evidence of higher rates of fluctuating cognitive impairment across regions, suggesting that this effect reflects differences in recognition of this feature, rather than differences in true prevalence.

Presence of visual hallucinations was associated with greater probability of receiving an LBD diagnosis in both the North East and London/South East. Multivariable analyses indicated that individuals without visual hallucinations had a low probability of receiving an LBD diagnosis in memory services outside North East England, suggesting that this is a key factor which decides whether individuals receive LBD diagnoses outside the North East. Presence of parkinsonism was associated with greater likelihood of receiving an LBD diagnosis without clear regional differences.

Presence of RBD was the strongest predictor of LBD diagnosis in North East England, but this was only weakly associated with diagnosis in London/South East, suggesting that this feature is not consistently ascertained or recognised in memory services in these regions, despite strong evidence linking RBD to Lewy pathology. There was tentative evidence of a higher rate of RBD reported by carers in North East England. However, this effect was not replicated by patient self-report and questions relating to RBD were the least consistently assessed using the toolkit. This requires replication and further investigation (eg, with polysomnography).

These findings suggest that regional differences in LBD diagnoses largely reflect differences in the recognition of LB clinical features. In particular, there are evident regional differences in the associations of cognitive fluctuations and RBD with LBD diagnosis. While visual hallucinations are strong positive predictors of LBD, it may be necessary to improve wider understanding that absence of visual hallucinations is not necessarily incompatible with LBD diagnosis because other features can be present supporting the diagnosis. Routine use of tools such as the DLB Assessment Toolkit, or searching electronic health records with natural language processing,^19^ might help identify clinical features relevant to diagnosis in healthcare settings. Such identification and improvement in accuracy of diagnosis could lead to important improvements in postdiagnostic care.

As previously noted,^2^ the North East of England has a long history of involvement in LBD research, which may lead to greater awareness of this condition in clinical education, training and research, as well as structural differences such as more routine use of DLB consensus criteria in memory services. Further research to assess practice-level and practitioner-level differences may clarify the specific causes of these differences in DLB diagnosis.

This analysis benefitted from a reasonably large sample of individuals recently diagnosed with dementia, from three different regional sites. Separate assessment with the DLB Assessment Toolkit and ascertainment of memory service LBD diagnoses enabled us to examine for the first time the possible reasons for regional differences in LBD diagnostic rates.

The differing application of diagnostic criteria across real-world services is central to this analysis and may explain regional differences in LBD diagnosis. Though NICE recommend McKeith criteria for differential diagnosis of DLB in specialist settings,^20^ it is unlikely that these are applied in a standardised way across all services. A recent audit of UK memory services indicated that 79 out of 125 services included (63%) implausibly reported no cases of DLB (while 112/125 reported no cases of frontotemporal dementia) suggesting that standardised criteria for dementia subtypes are not routinely administered across all services.^21^

The DLB Assessment Toolkit is intended to assist in standardised administration of the McKeith criteria, which has well-established diagnostic accuracy versus autopsy.^22^ Clinical services which introduced the DLB Assessment Toolkit saw an increase in DLB diagnoses, suggesting that this may improve the real-world sensitivity of existing criteria.^23^ However, the toolkit itself has not yet been validated versus autopsy.

This study also has five important limitations. First, LBD includes DLB and PDD, but these syndromes are diagnosed in different systems.^12 13^ In sensitivity analyses, we assessed possible differences between DLB and PDD diagnoses in this study, since the DLB Assessment Toolkit may be more sensitive to the former than the latter. In the North East and South East sites, recognised LBD cases were most often diagnosed with DLB (71% and 68%, respectively), whereas in London 100% of LBD cases were diagnosed with PDD. This could be an artefact of the relatively small LBD sample in London, or systematic differences in recognition or description of LBD across regions. However, these specific diagnoses did not appear to affect the observed findings, and the two subgroups did not differ in the number or pattern of clinical features endorsed in the toolkit.

Second, although the total sample size was reasonably large, completion rates for individual items in the toolkit varied, with missing data particularly common for items requiring the presence of a carer. The effective sample size is therefore lower than the total, although a strength of our analysis method enabled us to offset this limitation by the use of efficient Bayesian estimation methods and multiple imputation. This was a secondary analysis of existing data, and DETERMIND was not designed for this specific analysis and subgroup analyses, and so these may be subject to type II error.

Third, this study only covers three regions and so generalisability from these three regions may be limited. Regional differences have previously been reported across different memory services in the UK,^2^ but it is unclear whether these differences likewise reflect differences in recognition of the overall LBD clinical picture, specific DLB clinical features or true geographical differences in presence of LBD. We are also unable to extrapolate these findings outside UK memory services, though regional differences in PD prevalence have also been identified in other countries.^4 6^

Fourth, clinical diagnosis of LBD may be supported by the use of indicative biomarkers.^13^ Striatal dopaminergic imaging in particular is available to some services to improve recognition of LBD.^24^ We did not have access to data on indicative biomarkers during this study, and whether they were used in the services studied, so some of the observed inconsistencies between service diagnoses and research toolkit features might be explained by different rates of use of striatal dopaminergic imaging in different regions (ie, its use may be potentially higher in the North East). However, recent UK memory service audits indicate that despite the widespread availability of striatal dopaminergic imaging, this is infrequently used even in suspected DLB/PDD.^24^

Finally, this analysis is limited by the necessary algorithmic approach to DLB Assessment Toolkit. The toolkit is designed to be applied with clinical judgement for each item, which was not available in this study. Without detailed review of DLB clinical features and wider medical history, the toolkit will lack specificity in this study. There were notably high rates of fluctuating cognitive impairment identified when applying the recommended thresholds, suggesting a lack of specificity for these items in particular when expert clinical judgement is not incorporated. However, researchers who administered the DLB Assessment Toolkit received training from an expert clinician familiar with the development and implementation of this toolkit (AJT).

## Conclusions

UK regional differences in LBD diagnosis may be explained by differences in recognition of LBD diagnostic features. Absence of visual hallucinations may be seen as evidence against LBD leading to lack of diagnosis, while cognitive fluctuations and RBD may not be recognised. Appropriate use of the DLB Assessment Toolkit, or any similar tool, in healthcare services may help to address these diagnostic inequalities. Indications of regional differences in RBD rates may warrant further investigation.

## Supplementary material

10.1136/bmjopen-2026-121991 online supplemental file 1

## Data Availability

Data are available upon reasonable request.
