# Supplementary material for: Cross-sectional analysis of variation in diagnosis of Lewy body dementia in three English regions: data from the DETERMIND programme
Source: BMJ Open. 2026 Jul 22;16(7):e121991. doi: 10.1136/bmjopen-2026-121991 (PMC13404631; doi:10.1136/bmjopen-2026-121991)
Supplement: online supplemental file 1 [file bmjopen-16-7-s001.docx]

**Supplementary Table S1. Responses to individual screening questions from DLB Assessment Toolkit by site.**

|  | **Overall, N = 935***^1^* | **Gateshead, N = 258***^1^* | **London, N = 208***^1^* | **Sussex, N = 469***^1^* |
| --- | --- | --- | --- | --- |
| **Visual Hallucinations** |  |  |  |  |
| Does the patient have hallucinations such as seeing false visions? | 119 / 636 (19%) | 52 / 229 (23%) | 12 / 98 (12%) | 55 / 309 (18%) |
| Does [patient] seem to see things that are not present? | 111 / 636 (17%) | 50 / 228 (22%) | 11 / 98 (11%) | 50 / 310 (16%) |
| Do you feel like your eyes ever play tricks on you? | 219 / 828 (26%) | 79 / 247 (32%) | 49 / 184 (27%) | 91 / 397 (23%) |
| Have you ever seen something (or things) that other people could not see? | 147 / 833 (18%) | 54 / 252 (21%) | 26 / 184 (14%) | 67 / 397 (17%) |
| **Cognitive Fluctuations** |  |  |  |  |
| Does the patient show moderate changes in their level of functioning during the day? | 302 / 620 (49%) | 117 / 223 (52%) | 29 / 91 (32%) | 156 / 306 (51%) |
| Between getting up in the morning and going to bed at night, does the patient spend more than one hour sleeping? | 359 / 605 (59%) | 145 / 216 (67%) | 45 / 93 (48%) | 169 / 296 (57%) |
| Is the patient drowsy and lethargic for more than one hour during the day, despite getting their usual amount of sleep the night before? | 258 / 613 (42%) | 79 / 221 (36%) | 33 / 90 (37%) | 146 / 302 (48%) |
| Is it moderately difficult to arouse the patient so they maintain attention through the day? | 148 / 618 (24%) | 40 / 223 (18%) | 18 / 93 (19%) | 90 / 302 (30%) |
| **REM Sleep Behaviour Disorder** |  |  |  |  |
| Have you ever been told that you seem to “act out your dreams” while sleeping (punched or flailed arms in the air, shouted or screamed)? | 36 / 340 (11%) | 15 / 153 (9.8%) | 8 / 52 (15%) | 13 / 135 (9.6%) |
| Have you ever seen the patient appear to ‘‘act out his/her dreams’’ while sleeping (punched or flailed arms in the air, shouted or screamed)? | 68 / 277 (25%) | 23 / 73 (32%) | 10 / 48 (21%) | 35 / 156 (22%) |
| **Parkinsonism** |  |  |  |  |
| 5-Item UPDRS Score | 2.00 (4.00) | 0.00 (2.50) | 0.00 (2.00) | 3.00 (4.00) |
| *^1^* n / N (%); Median (IQR) | | | | |
