# Supplementary material for: Cross-sectional analysis of variation in diagnosis of Lewy body dementia in three English regions: data from the DETERMIND programme
Source: BMJ Open. 2026 Jul 22;16(7):e121991. doi: 10.1136/bmjopen-2026-121991 (PMC13404631; doi:10.1136/bmjopen-2026-121991)
Supplement: Supplementary file 2 [file bmjopen-2026-121991.reviewer_comments.pdf]

PEER REVIEW HISTORY

BMJ Open publishes all reviews undertaken for accepted manuscripts. Reviewers are asked to complete a checklist review form and are provided with free text boxes to elaborate on their assessment. These free text comments are reproduced below.

ARTICLE DETAILS

Title (Provisional)

Cross-sectional analysis of variation in diagnosis of Lewy body dementia in three English regions: data from the DETERMIND programme.

Authors

Hamilton, Calum Alexander; Thomas, Alan J.; Donaghy, Paul C; Hicks, Ben; Mueller, Christoph; Banerjee, Sube

---

VERSION 1 - REVIEW

**Reviewer**	**1**
**Name**	**Kim, Keun**
**Affiliation**	**Yonsei University, Department of Psychiatry**
**Date**	**04-Jun-2026**
**COI**	**None**

This study addresses an interesting topic regarding the regional disparities in the diagnosis of LBD within the UK. The findings highlight that the differences in diagnostic rates may stem from varying clinical recognition of core features rather than true prevalence. However, there are several issues that need to be addressed.

1. This study relies on real-world LBD diagnoses without specifying the diagnostic criteria used by clinicians (e.g., McKeith criteria). Please clarify if any standardized guidelines were applied across sites and discuss the resulting diagnostic variability as a limitation.

2. If any, the authors may better present the previously established diagnostic accuracy (sensitivity/specificity) of the DLB assessment toolkit. Please include prior validation metrics to ensure the accuracy of this method.

3. The Discussion attributes diagnostic disparities to differing clinician "awareness" and "recognition" but fails to explain why these differences exist. Please discuss potential structural reasons (e.g., disparities in local training).

4. Table 1 only reports descriptive statistics. Please add visual marks to clearly denote statistically significant differences between groups to ensure the table is stand-alone.

5. Tables and figures must be self-explanatory so that readers can fully understand the content without referring to the main text. In this context, please define all regional abbreviations in every single table or figure. Clarify in the Table 1 footnotes that 'Gateshead' represents the North East and 'Sussex' represents the South East. Additionally, explicitly define 'NE' and 'SE' in the Figure 1 caption.

**Reviewer**	**2**
**Name**	**Dack, Kyle**
**Affiliation**	**University of Bristol, MRC Integrative Epidemiology Unit**
**Date**	**08-Jun-2026**
**COI**	**None**

Statistical review:

The analysis and design are suitable for the aims and data, and the results are presented nicely. I have just a few minor suggestions regarding how the statistical analyses are described, and the interpretation of the assessment toolkit responses as indicative of underlying prevalence.

Abstract:

1. I suggest adding the study dates to the setting or another relevant section.

Methods:

2. the methods are clearly reported and align well with reporting guidelines. However, one aspect that could be elaborated on is that this appears to be essentially cross-sectional - with all data taken from baseline - from a larger prospective cohort study. This would be helpful to more directly state, because the reference to baseline may lead readers to expect some form of follow-up.

3. Analysis - I suggest some further details could be added to make this more transparent:

The software used, covariate selection rationale and adjustment, Bayesian model specifics (prior distribution, any sensitivity analyses, packages used), imputation details (variables added, assumed missing data mechanism, imputation parameters), and a full list of models. Listing all models would also be helpful so readers understand what is being added by each, and are able to interpret the results suitably.

Results:

4. The results are well presented, including tables and statistical content (no suggestions).

Discussion:

5. The results support the interpretation and I have no major comments. However, some of the interpretation may be overly strong, for example "The toolkit found similar rates of LB features across regions, indicating that there was little regional variation in actual LB disease rates" this is a plausible interpretation but does not directly demonstrate LB disease rates, as this could not be measured. Similarly, "Our findings suggest these differences in LBD prevalence were largely attributable to regional differences in the clinical recognition of LBD core clinical features" seems strong when what was measured is LB feature prevalence. The authors could consider softening this, or adding a rationale earlier in the discussion to support the assumed underlying prevalence from the toolkit features.

VERSION 1 - AUTHOR RESPONSE

Reviewer: 1

Author Response: We thank Dr Kim for their insightful review. We hope to have addressed their concerns in the below points.

1. This study relies on real-world LBD diagnoses without specifying the diagnostic criteria used by clinicians (e.g., McKeith criteria). Please clarify if any standardized guidelines were applied across sites and discuss the resulting diagnostic variability as a limitation.

Author Response: Thank you for this important point. As real-world diagnoses these are likely not standardised – indeed, this motivated our research question, and we believe standardised use of McKeith criteria would likely address the variable reporting of LBD across services. We have now clarified in the methods:

"As this leveraged real-world LBD diagnoses, the specific diagnostic criteria used (if any) were not known." (**Page 6, Lines 45-47**)

And also in the discussion:

"The differing application of diagnostic criteria across real-world services is central to this analysis, and may explain regional differences in LBD diagnosis. Though NICE recommend McKeith criteria for differential diagnosis of DLB in specialist settings, (20) it is unlikely that these are applied in a standardised way across all services. A recent audit of UK memory services indicated that 79 out of 125 services included (63%) implausibly reported no cases of DLB (while 112/125 reported no cases of frontotemporal dementia) suggesting that standardised criteria for dementia subtypes are not routinely administered across all services.(21)" (**Page 13, Line 40-50**)

2. If any, the authors may better present the previously established diagnostic accuracy

(sensitivity/specificity) of the DLB assessment toolkit. Please include prior validation metrics to ensure the accuracy of this method.

Author Response: Thank you for this important point. Related to the above point, we have now clarified:

"The DLB assessment toolkit is intended to assist in standardised administration of the McKeith criteria, which has well-established diagnostic accuracy vs autopsy.(22) Clinical services which introduced the DLB assessment toolkit saw an increase in DLB diagnoses, suggesting that this may improve the real-world sensitivity of existing criteria.(23) However, the toolkit itself has not yet been validated vs. autopsy." (**Page 13, Line 51 to Page 14, Line 5**)

3. The Discussion attributes diagnostic disparities to differing clinician "awareness" and "recognition" but fails to explain why these differences exist. Please discuss potential structural reasons (e.g., disparities in local training).

Author Response: Thank you for this important point. The North East of England (Newcastle University in particular) has played a prominent role in DLB research from the first consensus criteria to the development of the DLB Assessment Toolkit. DLB features prominently in our (bio)medical education and research, and practicing clinicians in the North East are likely more familiar with the condition as a result. As subjective observations, we are unable to evidence this inherently speculative point, however, we hope to have clarified:

"As previously noted,(2) the North East of England has a long history of involvement in LBD research, which may lead to greater awareness of this condition in clinical education, training and research, as well as structural differences such as more routine use of DLB consensus criteria in memory services. Further research to assess practice- and practitioner-level differences may clarify the specific causes of these differences in DLB diagnosis." (**Page 13, Lines 27-33**)

4. Table 1 only reports descriptive statistics. Please add visual marks to clearly denote statistically significant differences between groups to ensure the table is stand-alone.

Author Response: Thank you for this point. We have now provided results of frequentist omnibus statistical tests to Table 1 as requested. (**Page 9, Table 1**)

5. Tables and figures must be self-explanatory so that readers can fully understand the content without referring to the main text. In this context, please define all regional abbreviations in every single table or figure. Clarify in the Table 1 footnotes that 'Gateshead' represents the North East and 'Sussex' represents the South East. Additionally, explicitly define 'NE' and 'SE' in the Figure 1 caption.

Author Response: Thank you for raising this oversight, and we apologise for the lack of clarity. We have revised our geographical descriptions in both Table 1 and Figure 1 to

make this clear. **(Page 9, Table 1 & Figure 1**)

Reviewer: 2

Author Response: We thank Dr Dack for their fair and actionable points. We hope we have addressed these clearly.

1. I suggest adding the study dates to the setting or another relevant section.

Author Response: Thank you for identifying this oversight. We have now amended the abstract setting as suggested:

"Memory clinics in three sites across England (North East, London, South East) from July 2019 to March 2023" (**Page 2, Lines 19-21**)

2. the methods are clearly reported and align well with reporting guidelines. However, one aspect that could be elaborated on is that this appears to be essentially cross-sectional - with all data taken from baseline - from a larger prospective cohort study. This would be helpful to more directly state, because the reference to baseline may lead readers to expect some form of follow-up.

Author Response: Thank you for this point. We have now clarified in the methods:

"We conducted a cross-sectional analysis of baseline data from an ongoing longitudinal study of people recently diagnosed with dementia." (**Page 6, Lines 5-8**)

3. Analysis - I suggest some further details could be added to make this more transparent: The software used, covariate selection rationale and adjustment, Bayesian model specifics (prior distribution, any sensitivity analyses, packages used), imputation details (variables added, assumed missing data mechanism, imputation parameters), and a full list of models. Listing all models would also be helpful so readers understand what is being added by each, and are able to interpret the results suitably.

Author Response: Thank you for identifying this need. We have expanded our analysis description as suggested:

"All analyses were conducted using *R* statistical software for Windows (Version 4.5.0) with the *brms* package as an interface to the *Stan* probabilistic programming language. Age and gender were included as covariates to account for possible systematic differences and the known male/female imbalance in DLB diagnoses.

LBD diagnostic rates were assessed with a Bayesian binomial probit model (codifying relationships with a binary variable in a comparable manner to a frequentist logistic regression, and enabling features to be treated as ordinal predictors), including the four core clinical features specifically or total number of core features endorsed as

predictors, with a binary site (North East vs London/South East, in the absence of any hypothesised London vs South East difference) interaction. Individual core clinical features were included as binary predictors, while the total number of features endorsed was treated as a monotonic ordinal variable.

Primary analyses were conducted with listwise deletion for missing data. Sensitivity analyses were also conducted with missing data multiply imputed using the *mice* package with predictive mean matching. All analytical variables (LBD diagnosis, region, age, gender, and the four core clinical features of DLB) were included with an assumption that missingness was non-informative (i.e. missing at random or missing completely at random, for example reflecting unavailability of an informant which was more common for women). Posterior distributions were pooled by Bayesian methods.

Strength of evidence for each effect was interpreted with reference to the corresponding Evidence Ratio (ER; the weight of evidence for a given hypothesis:the weight of evidence against this) for each effect, equivalent to a Bayes Factor (BF) for a directed hypothesis.(18) ER values > 1 reflect evidence in favour of the hypothesis, values < 1 reflect more evidence against the hypothesis, while values ≈ 1 reflect equivocal evidence. Bayesian analyses included weakly informative Student T priors with intercepts anticipating that LBD would represent a minority of dementia cases ($v = 3$, $\mu = -2$, $\sigma = 1$), and zero-centred coefficients for all predictor slopes ($v = 8$, $\mu = 0$, $\sigma = 1$). Prior predictive checks and prior sensitivity analyses were used to check the robustness of results with different prior specifications.” (**Page 7 Line 41 to Page 8 Line 26**)

4. The results are well presented, including tables and statistical content (no suggestions).

Author Response: Thank you for this appraisal (no changes required).

5. The results support the interpretation and I have no major comments. However, some of the interpretation may be overly strong, for example "The toolkit found similar rates of LB features across regions, indicating that there was little regional variation in actual LB disease rates" this is a plausible interpretation but does not directly demonstrate LB disease rates, as this could not be measured. Similarly, "Our findings suggest these differences in LBD prevalence were largely attributable to regional differences in the clinical recognition of LBD core clinical features" seems strong when what was measured is LB feature prevalence. The authors could consider softening this, or adding a rationale earlier in the discussion to support the assumed underlying prevalence from the toolkit features.

Author Response: Thank you for this point. We have amended the language to soften the conclusions and hopefully better reflect the uncertainty in the data to hand:

“The toolkit found similar rates of LB features across regions, which did not clearly support theorised regional differences in actual LB disease rates” (**Page 12, Lines 17-19**)

“Our findings were therefore most consistent with these differences in LBD diagnoses reflecting regional differences in the clinical recognition of LBD core clinical features” (**Page 12, Lines 24-26**)
